# Is there a place for Psychosocial Rehabilitation in Alcohol Use Disorders? – A Retrospective Analysis of the profile of frequent Users of an Inpatient Alcohol Unit in Lisbon

**DOI:** 10.1192/j.eurpsy.2025.925

**Published:** 2025-08-26

**Authors:** M. Andrade, M. Cameira, I. Pereira, A. Ferreira, S. Morais, J. Teixeira

**Affiliations:** 1 Hospital Júlio de Matos - Unidade Local de Saúde São José, Lisbon; 2 Hospital de Setúbal, Setúbal; 3 Hospital Garcia de Orta, Almada, Portugal

## Abstract

**Introduction:**

Alcohol dependence is a chronic condition associated with multiple relapses, leading to recurrent admissions to inpatient units. The success of treatment is closely tied to the psychosocial rehabilitation of these patients, as a means to ensure long-term abstinence.

**Objectives:**

We aim to characterize the psychosocial profile of frequent users of an Inpatient Alcohol Detoxification Unit in Lisbon and to reflect on the need for psychosocial interventions to prevent relapse risk.

**Methods:**

A retrospective analysis of data collected from the clinical records of patients admitted two or more times within one year to an Alcohol Detoxification Unit in Lisbon, during the period between January 2022 and December 2023.

**Results:**

During the study period, 37 patients with two or more admissions in a year were identified. The average age was 51.9 years, and 67.6% were male. It was found that 48.6% of the patients were divorced or separated; more than half of the patients were unemployed at the time of admission (62.2%), and nearly half were experiencing financial hardship (48.6%). In terms of integration into rehabilitative and abstinence maintenance structures, 40.5% had attended the Day Care Center of the Hospital Center, and only 5.4% had been part of a Therapeutic Community (TC). Before their last admission, 8.1% of the patients had been referred to a TC, 10.8% to the Day Care Center, and 51.4% to outpatient care, while 24.3% left against medical advice. In contrast, during the last admission in the study period, 13.5% were referred to the Day Care Center, and 35.1% to a TC.

**Conclusions:**

The results highlight the need for psychosocial intervention and rehabilitation in patients with alcohol use disorder. Treatment should include a multidisciplinary approach that takes into account socioeconomic support and integration into rehabilitative structures, as these promote long-term abstinence and therapeutic success.

**Disclosure of Interest:**

None Declared

